# Hungry for connection: associations between social isolation, mental health, and food insecurity in regional Australian adults

**DOI:** 10.1093/heapro/daaf176

**Published:** 2025-10-24

**Authors:** Katherine Kent, Alemayehu Digssie Gebremariam, Denis Visentin, Kelly Andrews, Grace Potter, Karen Charlton

**Affiliations:** School of Medical, Indigenous and Health Sciences, University of Wollongong, Wollongong, NSW 2522, Australia; School of Medical, Indigenous and Health Sciences, University of Wollongong, Wollongong, NSW 2522, Australia; School of Health Sciences, University of Tasmania, Launceston, TAS 7250, Australia; Healthy Cities Australia, Wollongong, NSW 2500, Australia; Healthy Cities Australia, Wollongong, NSW 2500, Australia; School of Medical, Indigenous and Health Sciences, University of Wollongong, Wollongong, NSW 2522, Australia

**Keywords:** food insecurity, mental health, social isolation, regional Australia, social determinants of health, public health nutrition

## Abstract

Food insecurity is increasingly recognized as a public health issue with social and psychological dimensions. However, evidence on its association with mental health and social isolation in regional Australia remains limited. A cross-sectional online survey among adults (≥18 years) living in the Illawarra and Shoalhaven regions of Australia assessed food insecurity using the 18-item USDA Household Food Security Survey Module, categorized into ‘food secure’ (score = 0) or ‘food insecure’ (score = 1+). Self-rated mental health and physical health (excellent/good vs. fair/poor) and reported diagnosis of a mental health condition was determined. Social isolation was assessed using six individual indicators and a composite social isolation score dichotomized participants into high versus low isolation. Multivariate logistic regression models examined associations between mental health, social isolation, and food insecurity, adjusting for relevant sociodemographic characteristics. Of participants (*n* = 666; 80% female; 57.1% university education), 38.3% experienced food insecurity. Poor self-rated mental health (22%) was associated with increased odds of food insecurity (AOR = 2.41, 95% CI: 1.47–3.96), as was diagnosed mental illness (AOR = 2.33, 95% CI: 1.46–3.71) and poor self-rated physical health (AOR = 2.06, 95% CI: 1.23–3.44). Participants with high social isolation on the composite score (15.5%) had two times higher odds of food insecurity compared with those with low isolation (AOR = 2.16, 95% CI: 1.09–4.26). Strong associations were also observed for individual indicators. Findings demonstrate strong links between food insecurity, mental health, and social isolation in regional Australia. Addressing food insecurity requires integrated strategies that combine material assistance with initiatives to strengthen social connectedness.

Contribution to Health Promotion

- Provides the first empirical evidence from regional Australia linking food insecurity with poor mental health and diagnosed mental illness.
- Demonstrates that multiple forms of social isolation (e.g. feeling friendless, lacking support, community exclusion) significantly increase food insecurity risk.
- Generates critical regional evidence to support advocacy for integrated food system and social connection strategies tailored to regional Australian contexts.

## INTRODUCTION

Food insecurity is defined as the ‘limited or uncertain availability of nutritious, safe foods—or the inability to acquire them in socially acceptable ways’ due to financial, physical, or social constraints (Gallegos *et al*. 2023). It encompasses a spectrum ranging from anxiety over food access to actual experiences of hunger and disrupted eating patterns. It is increasingly recognized as a key social determinant of health, with implications that extend beyond nutrition to impact physical, mental, and social wellbeing (Compton 2014). In Australia, estimates suggest that approximately one in three adults (32%) experience some level of food insecurity (Foodbank Australia 2023). Rates are consistently higher among groups facing social disadvantage, including low-income households, Aboriginal and Torres Strait Islander peoples, single-parent families, and people from culturally and linguistically diverse (CALD) backgrounds (Pollard and Booth 2019). Further, people living in regional and rural areas often experience higher rates of food insecurity (Kent *et al*. 2022) due to compounding factors such as limited food access, lower income, housing insecurity, geographical isolation, and reduced availability of support services (Mungai *et al*. 2020).

A substantial international evidence base has established strong associations between food insecurity and a range of adverse health outcomes (Gundersen and Ziliak 2015). These include increased risk of chronic physical conditions (Weaver and Fasel 2018) such as diabetes, cardiovascular disease, and obesity, poor self-rated physical health (Lee 2022), and increased healthcare utilization (Men *et al*. 2020). A growing body of literature also identifies robust links between food insecurity and mental health conditions including self-rated mental health (Pourmotabbed *et al*. 2020) and diagnoses of depression, anxiety, and psychological distress (Jones 2017). These associations are thought to be bidirectional: the stress and uncertainty of accessing food can negatively affect mental health, while poor mental health may limit individuals’ capacity to work, manage finances, or access food support systems (Hill *et al*. 2023).

Related to mental health outcomes, food insecurity is also increasingly linked to social isolation and reduced sense of community belonging (Martin *et al*. 2016). International studies show that this relationship is also mutually reinforcing, where social isolation can increase vulnerability to food insecurity by limiting access to informal food-sharing networks, transport, and emotional support, while food insecurity can contribute to withdrawal from social participation due to stigma, shame, and financial strain (Park and Berkowitz 2024). Individuals experiencing food insecurity are more likely to report feeling alone, excluded, or unsupported, which may further exacerbate the psychological burden of food insecurity and limit access to social networks and informal support (Park and Berkowitz 2024). The co-occurrence of food insecurity and social isolation has been associated with poorer quality of life, greater healthcare utilization, and, in some studies, increased suicidal ideation (Gonyea *et al*. 2022, Graham and Ciciurkaite 2023). These experiences are particularly concerning in regional Australian contexts, where access to health, social, and food services is often more limited (Barton *et al*. 2024). Despite this growing body of research internationally, there is a lack of local-level data on food insecurity and its associated predictors in Australia to inform regionally targeted public health interventions. National surveys rarely capture the nuanced experiences of food insecurity in regional communities. Without locally relevant data, it is difficult for regional health services and policymakers to design targeted interventions or advocate for place-based solutions that address the interconnected impacts of food insecurity on mental health, physical health and social isolation. Therefore, the aim of this study was to examine the associations between household food insecurity and self-reported physical and mental health outcomes, including indicators of social isolation, in a regional Australian adult population.

## METHODS

### Setting

This study was conducted in the Illawarra Shoalhaven region of New South Wales, Australia which is a geographically and socioeconomically diverse area comprising urban, coastal, and rural communities south of Sydney. The region faces ongoing challenges related to food access and health inequality, particularly in disadvantaged areas. The Let’s Talk About Food survey was a community-driven initiative developed in partnership with local organizations (University of Wollongong in partnership with Healthy Cities Australia and Food Fairness Illawarra) to capture residents’ experiences of food access, health, and wellbeing. As a regional case study, it offers insights relevant to other areas facing similar structural barriers to food security.

### Study design and participants

A cross-sectional online survey was conducted between April and May 2024. Recruitment was conducted using a combination of convenience sampling (social media, email networks, posters/flyers in community organizations) and a targeted flyer mailout to households across the region. The targeted mailout was conducted using a stratified random sample of 10 000 residential addresses across the four Illawarra Shoalhaven LGAs (Kiama, Shellharbour, Shoalhaven, Wollongong), proportional to the adult population in each area. Addresses were drawn from the Geocoded National Address File, restricted to residential land use zones, and issued with unique access codes to enable secure survey participation. Eligible participants were adults (≥18 years) residing in the region. All participants provided informed consent prior to commencing the survey. Ethics approval was obtained from the University of Wollongong Human Research Ethics Committee (approval number 2024/030).

### Food insecurity

Household food insecurity was assessed using the validated 18-item Household Food Security Survey Module (HFSSM), comprising 10 adult-referenced and 8 child-referenced items (Bickel *et al*. 2000). These questions captured experiences of food-related hardship due to financial constraints over the past 30 days. Responses were coded as affirmative based on standard USDA criteria. Then, the classification system used by PROOF Canada and Health Canada was applied where a household was considered food secure if no items were affirmed. Marginal food insecurity was defined as one affirmative response on either the adult or child scale. Moderate food insecurity was classified as two to five affirmative responses on the adult scale or two to four on the child scale. Severe food insecurity was defined as six or more affirmative responses on the adult scale or five or more on the child scale. In households with children, both adult and child items were used in scoring, and if classifications differed, the more severe status was assigned. For regression analyses, food insecurity was treated as a binary variable, with households classified as food secure (zero affirmative responses) or food insecure (one or more affirmative responses).

### Self-reported health outcomes

Participants were asked to rate their overall physical and mental health using the validated questions (Lundberg and Manderbacka 1996): ‘In general, how would you rate your physical health?’ and ‘In general, how would you rate your mental health?’ Response options ranged from Excellent to Poor and were collapsed into two categories for analysis: Excellent/Very Good/Good versus Fair/Poor (Bombak 2013). Participants also reported whether they had been diagnosed with mental health issues (including depression or anxiety; yes/no). These single-item self-rated health questions are widely used in population health research and are considered reliable indicators of general health status (Bombak 2013).

Social isolation was assessed using a series of six items asking participants how often they experienced specific social feelings or situations (It has been easy to relate to others; I had someone to share my feelings with; I found it easy to get in touch with others when I needed to; I felt isolated from other people; When with other people, I felt separate from them; I felt alone and friendless). Response options were coded on a five-point Likert scale: 1 = Almost always, 2 = Most of the time, 3 = About half of the time, 4 = Occasionally, and 5 = Not at all. To create a composite score, positively worded items were reverse-coded, and an overall composite score was calculated by averaging scores across all six sub scales, with lower scores indicating higher social isolation. Internal consistency for the scales were high (Cronbach’s *α* = 0.879), exceeding the conventional threshold of 0.70 for acceptable reliability (Terwee *et al*. 2007). A binary classification was then created, where scores of 1–3 were categorized as high social isolation (indicating frequent feelings of disconnection or low connectedness), while scores of 4–5 were categorized as low social isolation.

### Sociodemographic variables

Participants provided information on gender (male, female, other), education level (primary/secondary school, diploma/Technical and Further Education (TAFE), university), Aboriginal and/or Torres Strait Islander status (yes/no), country of birth (Australia vs overseas), household composition (living alone, couple with or without dependents, single parent, other), employment status (employed, unemployed, retired, student, other), presence of a health condition or disability (yes/no), using a language other than English at home and annual household income (<$26 000; $26 000–51 999; $52 000–77 999; $78 000–103 999; $104 000–155 999; >$156 000; prefer not to say).

### Data analysis

Of the 1014 individuals who began the survey, 666 (65.7%) completed the USDA HFSSM and were included in the final analysis. Completion rates varied by recruitment method: 72 out of 160 from the postal mailout (45.0%), 539 out of 792 from social media (68.1%), and 55 out of 62 from community-based recruitment (88.7%). Data cleaning involved excluding incomplete surveys and checking for implausible responses; no data imputation was performed. Descriptive statistics were used to summarize participant characteristics, food security status, self-reported health and social isolation outcomes. Pearson’s correlation coefficients were also calculated to explore the strength and direction of bivariate relationships between food insecurity, health indicators, and social isolation variables. Logistic regression models were used to examine the association between food insecurity, self-reported health outcomes (poor-fair physical health, poor-fair mental health (Plante *et al*. 2024) and diagnosed mental health condition) and social connection and perceived support, adjusting for relevant sociodemographic variables. Covariates included in regression analyses were selected *a priori* based on previous literature and theoretical relevance (age, education, Aboriginal and/or Torres Strait Islander status, disability, language other than English at home, country of birth, household composition, employment status, and total pre-tax income). Adjusted odds ratios (AORs) and 95% confidence intervals (CIs) were reported. Data were assessed for multicollinearity using variance inflation factors (VIFs) and tolerance, with all VIFs below 2 and tolerance < 0.2, indicating no evidence of collinearity between predictors. Participants with missing data for exposure, outcome, or covariates in regression analyses were excluded using complete case analysis; no imputation was performed. All analyses were conducted using SPSS Version: 28.0.1.0 (142).

## RESULTS

Table 1 presents the demographic profile of survey respondents (*n* = 666). Most respondents identified as female (*n* = 526, 79.8%), with 18.8% (*n* = 124) identifying as male and 1.4% (*n* = 9) as another gender. The largest age group was 45–54 years (*n* = 125, 19.0%), followed by 35–44 years (*n* = 123, 18.7%) and 55–64 years (*n* = 119, 18.1%). Most participants held a university qualification (*n* = 338, 57.1%), while 28.4% (*n* = 168) had a diploma or TAFE qualification, and 14.5% (*n* = 86) had completed only primary or secondary education. A small proportion of the sample identified as Aboriginal and/or Torres Strait Islander (*n* = 18, 2.7%). In terms of household composition, 36.8% (*n* = 245) lived as a couple without dependents, 27.4% (*n* = 182) as a couple with dependents, 21.7% (*n* = 144) lived alone, and 7.7% (*n* = 51) were single parents. Over half (*n* = 386, 58.6%) were employed, while 21.4% (*n* = 141, were retired, 7.3% (*n* = 48) unemployed, and 5.3% were students (*n* = 35). Nearly one-third (*n* = 196, 29.5%) reported having a health condition or disability that limited activity. Household income varied, with 20.0% (*n* = 130) earning $26 000–$51,999, 19.2% (*n* = 125) earning $52 000–$77,999, and 18.9% (*n* = 123) earning $78 000–$103 999 annually.

**Table 1.** Demographic characteristics of survey participants (*N* = 666).

	Total sample	Food secure	Food insecure
Characteristic	*N* (%)	*N* (%)	*N* (%)
Age			
18–24 years	41 (6.2%)	16 (39.0%)	25 (61.0%)
25–34 years	106 (16.1%)	49 (46.2%)	57 (53.8%)
35–44 years	123 (18.7%)	65 (52.8%)	58 (47.2%)
45–54 years	125 (19.0%)	77 (61.6%)	48 (38.4%)
55–64 years	119 (18.1%)	94 (79.0%)	25 (21.0%)
65–74 years	104 (15.8%)	76 (73.1%)	28 (26.9%)
75 years or older	39 (5.9%)	28 (71.8%)	11 (28.2%)
Gender			
Male	124 (18.8%)	67 (54.0%)	57 (46.0%)
Female	526 (79.8%)	335 (63.7%)	191 (36.3%)
Other	9 (1.4%)	4 (44.4%)	5 (55.6%)
Education			
Primary/secondary	86 (14.5%)	41 (47.7%)	45 (52.3%)
Diploma/TAFE	168 (28.4%)	87 (51.8%)	81 (48.2%)
University	338 (57.1%)	237 (70.1%)	101 (29.9%)
Aboriginal or Torres Strait Islander			
Yes	18 (2.7%)	1 (5.6%)	17 (94.4%)
No	640 (97.3%)	404 (63.1%)	236 (36.9%)
Household composition			
Living alone	144 (21.7%)	76 (52.8%)	68 (47.2%)
Couple no dependents	245 (36.8%)	185 (75.5%)	60 (24.5%)
Couple with dependents	182 (27.4%)	114 (62.6%)	68 (37.4%)
Single parent	51 (7.7%)	17 (33.3%)	34 (66.7%)
Other	43 (6.5%)	18 (41.9%)	25 (58.1%)
Employment status			
Employed	386 (58.6%)	249 (64.5%)	137 (35.5%)
Unemployed	48 (7.3%)	16 (33.3%)	32 (66.7%)
Retired	141 (21.4%)	108 (76.6%)	33 (23.4%)
Student	35 (5.3%)	17 (48.6%)	18 (51.4%)
Other	49 (7.4%)	18 (36.7%)	31 (63.3%)
Health condition or disability			
Yes	196 (29.5%)	99 (50.5%)	97 (49.5%)
No	469 (70.5%)	312 (66.5%)	157 (33.5%)
Annual household income			
<26 000			
26,000–51 999	130 (20.0%)	58 (44.6%)	72 (55.4%)
52,000–77 999	125 (19.2%)	68 (54.4%)	57 (45.6%)
78 000–103 999	123 (18.9%)	75 (61.0%)	48 (39.0%)
104 000–155 999	91 (14.0%)	62 (68.1%)	29 (31.9%)
>156 000	94 (14.4%)	78 (83.0%)	16 (17.0%)
Prefer not to say	33 (5.1%)	29 (87.9%)	4 (12.1%)

Of the total sample, 38.3% (*n* = 255) were classified as food insecure compromising 7.8% (*n* = 52) experiencing marginal food insecurity, 18.8% (*n* = 125) moderate food insecurity, and 11.7% (*n* = 78) severe food insecurity. Higher proportions of food insecurity were identified among those aged 18–24 years (*n* = 25; 61.0%) and 25–34 years (*n* = 57, 53.8%) compared to 21.0% (*n* = 25) among those aged 55–64 years (Table 1). More than half (*n* = 45, 52.3%) of those with primary or secondary education reported food insecurity compared to 29.9% (*n* = 101) of university-educated participants. The proportion of food insecurity was also higher among Aboriginal or Torres Strait Islander participants (*n* = 17, 94.4%) compared to non-Indigenous participants (*n* = 236, 36.9%), and among those with a health condition or disability (*n* = 97, 49.5% vs. *n* = 157, 33.5%). Higher proportions of single parents (*n* = 34, 66.7%) and individuals living alone (*n* = 68, 47.2%) reported food insecurity compared to couples without dependents (*n* = 60, 24.5%). Higher proportions of food insecurity were reported among unemployed participants (*n* = 34, 66.7%) and those in ‘other’ categories (*n* = 31, 63.3%) than among retirees (*n* = 33, 23.4%). The proportion of food insecurity varied by household income with over half (*n* = 72, 55.4%) of participants earning $26 000–$51 999 and 45.6% (*n* = 57) of those earning $52 000–$77 999 reporting food insecurity, compared to 31.9% (*n* = 29) of those earning $104 000–$155 999 and 17.0% (*n* = 16) of those earning over $156 000.

Most respondents rated their physical (*n* = 486, 77.7%) and mental health (*n* = 488, 78.1%) as good, very good, or excellent (Table 2). However, 16.0% (*n* = 100) reported fair and 6.2% (*n* = 39) poor physical health, while 15.4% (*n* = 96) reported fair and 6.6% (*n* = 41) poor mental health. Over one-quarter (*n* = 157, 27.7%) reported a diagnosed mental health condition. Social connection indicators varied. While 36.8% (*n* = 229) reported that yes, they felt part of their local community and a further 44.6% (*n* = 278) said they did sometimes. However, 15.7% (*n* = 98) did not have a sense of belonging. Based on the composite score, 15.5% (*n* = 95) of participants were classified as experiencing high social isolation across the six-subscales. Just over 70% (*n* = 451) reported it was easy to relate to others at least most of the time, and 75.9% (*n* = 474) had someone to share their feelings with at least most of the time. However, 23.7% (*n* = 148) felt isolated from others about half the time or more, and 16.7% (*n* = 101) reported often feeling alone and friendless. Perceived separateness in social settings was also common, with 20.1% (*n* = 125) reporting this feeling at least about half the time. Some participants (*n* = 90, 14.4%) reported difficulty getting in touch with others when needed.

**Table 2.** Self-rated health, and perceptions of social connection and community belonging among respondents.

Variable	Response option	*n* (%)
Self-rated physical health (*n* = 625)	Excellent	64 (10.2%)
	Very good	212 (33.9%)
	Good	210 (33.6%)
	Fair	100 (16.0%)
	Poor	39 (6.2%)
Self-rated mental health (*n* = 625)	Excellent	66 (10.6%)
	Very good	204 (32.6%)
	Good	218 (34.9%)
	Fair	96 (15.4%)
	Poor	41 (6.6%)
Self-reported diagnosis of mental health condition (*n* = 566)	Yes	157 (27.7%)
	No or not ever diagnosed	409 (72.3%)
Do you feel part of your local community (*n* = 623)	Yes	229 (36.8%)
	Sometimes	278 (44.6%)
	No	98 (15.7%)
	I don’t know	18 (2.9%)
Composite score for social isolation (*n* = 611)	Higher social isolation	95 (15.5%)
	Lower social isolation	516 (84.5%)
It has been easy to relate to others (*n* = 628)	Almost always	189 (30.1%)
	Most of the time	262 (41.7%)
	About half of the time	96 (15.3%)
	Occasionally	67 (10.7%)
	Not at all	14 (2.2%)
I had someone to share my feelings with (*n* = 624)	Almost always	296 (47.4%)
	Most of the time	178 (28.5%)
	About half of the time	55 (8.8%)
	Occasionally	71 (11.4%)
	Not at all	24 (3.8%)
I felt isolated from other people (*n* = 624)	Almost always	20 (3.2%)
	Most of the time	47 (7.5%)
	About half of the time	81 (13.0%)
	Occasionally	251 (40.2%)
	Not at all	225 (36.1%)
I found it easy to get in touch with others when I needed to (*n* = 624)	Almost always	241 (38.6%)
	Most of the time	217 (34.8%)
	About half of the time	76 (12.2%)
	Occasionally	70 (11.2%)
	Not at all	20 (3.2%)
When with other people, I felt separate from them (*n* = 622)	Almost always	17 (2.7%)
	Most of the time	36 (5.8%)
	About half of the time	72 (11.6%)
	Occasionally	247 (39.7%)
	Not at all	250 (40.2%)
I felt alone and friendless (*n* = 625)	Almost always	24 (3.8%)
	Most of the time	26 (4.2%)
	About half of the time	51 (8.2%)
	Occasionally	165 (26.4%)
	Not at all	359 (57.4%)

**Figure 1.**
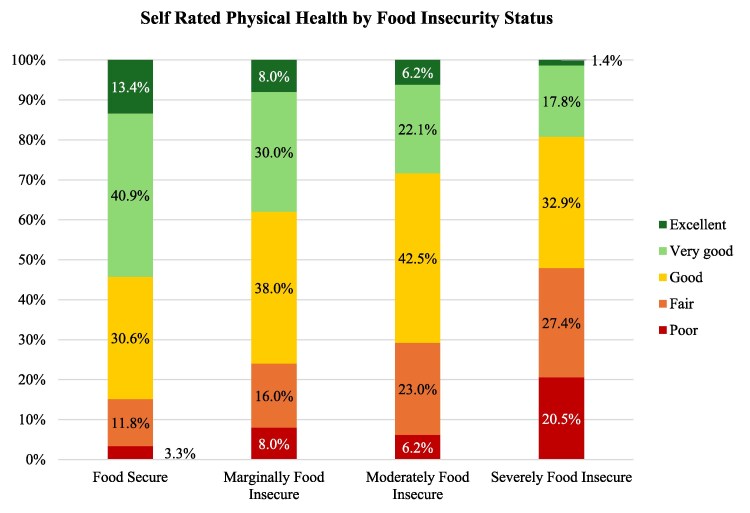
Self-rated health physical health (Excellent, Very Good, Good, Fair, Poor) by food security categories (*n* = 625).

**Figure 2.**
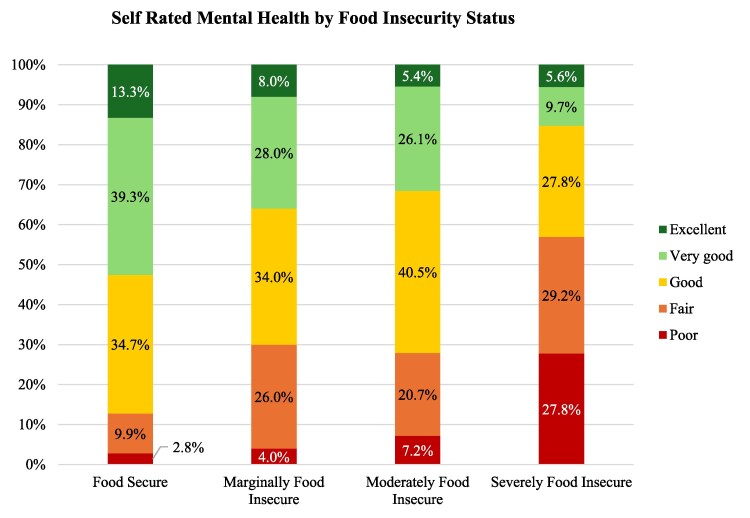
Self-rated health mental health (Excellent, Very Good, Good, Fair, Poor) by food security categories (*n* = 625).

As shown in Figs 1 and 2, the proportion of participants reporting fair or poor health increased markedly with food insecurity severity. For physical health, fair/poor ratings rose from 15.6% among food secure adults to 47.9% among those who were severely food insecure. A similar gradient was observed for mental health, with fair/poor ratings increasing from 12.4% in the food secure group to 57.0% in the severely food insecure group. As shown in Table 3, participants who rated their physical health as fair or poor had significantly greater odds of experiencing food insecurity compared to those reporting excellent or good health (AOR = 2.06; 95% CI: 1.23–3.44). Similarly, participants with fair or poor mental health had over twice the odds of food insecurity (AOR = 2.41; 95% CI: 1.47–3.96), as did those with a diagnosed mental health condition (AOR = 2.33; 95% CI: 1.46–3.71). Correlation analysis (Supplementary Table S1) showed a moderate positive association between poorer physical health and food insecurity (*r* = 0.32, *P* < 0.01), and physical health was also strongly related to mental health (*r* = 0.57, *P* < 0.01).

**Table 3.** Associations between social connectedness, mental and physical health, and food insecurity status among adults.

	Food secure *n* (%)	Food insecure *n* (%)	OR	SE	95%CI	*P*-value	AOR	SE	95%CI	*P*-value
**Self-rated physical health**										
Excellent/good	330 (67.9%)	156 (32.1%)	REF				REF			
Fair/poor	59 (42.4%)	80 (57.6%)	2.87	0.20	[1.95, 4.22]	<0.001	2.06	0.26	[1.23, 3.44]	0.006
**Self-rated mental health**										
Excellent/good	342 (70.1%)	146 (29.9%)	REF				REF			
Fair/poor	50 (36.5%)	87 (63.5%)	4.08	0.20	[2.74, 6.07]	<0.001	2.41	0.25	[1.47, 3.96]	<0.001
**Diagnosed mental health issue**										
Yes	65 (41.4%)	92 (58.6%)	3.25	0.20	[2.22, 4.76]	<0.001	2.33	0.24	[1.46, 3.71]	<0.001
No	285 (69.7%)	124 (30.3%)	REF				REF			
**Do you feel part of your local community**										
Yes—sometimes	331 (65.3%)	176 (34.7%)	REF				REF			
No	46 (46.9%)	52 (53.1%)	2.13	0.22	[1.37, 3.29]	<0.001	1.87	0.28	[1.08, 3.26]	0.026
**Composite Social Isolation Score**										
High social isolation	350 (67.8%)	166 (32.2%)	REF				REF			
Low social isolation	33 (34.7%0)	62 (65.3%)	3.96	0.24	[2.50, 6.28]	<0.001	2.16	0.35	[1.09, 4.26]	0.027
**It has been easy to relate to others**										
Almost always—about half of the time	357 (65.3%)	190 (34.7%)	REF				REF			
Occasionally—not at all	35 (43.2%)	46 (56.8%)	2.47	0.24	[1.59, 3.97]	<0.001	1.64	0.30	[0.90, 2.99]	0.103
**I had someone to share my feelings with**										
Almost always—about half of the time	345 (65.2%)	184 (34.8%)	REF				REF			
Occasionally—not at all	43 (45.3%)	52 (54.7%)	2.27	0.23	[1.46, 3.53]	<0.001	2.41	0.29	[1.37, 4.24]	0.002
**I felt isolated from other people**										
Almost always—about half of the time	58 (39.2%)	90 (60.8%)	3.58	0.20	[2.44–5.25]	<0.001	2.91	0.24	[1.81, 4.67]	<0.001
Occasionally—not at all	332 (69.7%)	144 (30.3%)	REF				REF			
**I found it easy to get in touch with others when I needed to**										
Almost always—about half of the time	351 (65.7%)	183 (34.3%)	REF				REF			
Occasionally—not at all	39 (43.3%)	51 (56.7%)	2.51	0.21	[1.59, 3.95]	<0.001	1.99	0.28	[1.14, 3.46]	0.015
**When with other people, I felt separate from them**										
Almost always—about half of the time	47 (37.6%)	78 (62.4%)	3.70	0.21	[2.46–5.56]	<0.001	2.73	0.26	[1.65, 4.52]	<0.001
Occasionally—not at all	343 (69.0%)	154 (31.0%)	REF				REF			
**I felt alone and friendless**										
Almost always—about half of the time	34 (33.7%)	67 (66.3%)	4.17	0.23	[2.66–6.56]	<0.001	3.13	0.28	[1.81, 5.43]	<0.001
Occasionally—not at all	356 (67.9%)	168 (32.1%)	REF				REF			

OR, crude (unadjusted) odds ratio; AOR, adjusted odds ratio Adjusted models control for age, education, Aboriginal and/or Torres Strait Islander status, activity-limiting disability, country of birth, language spoken at home, household type, employment status, and total pre-tax income; SE, standard error; 95% CI, 95% confidence interval; REF, reference group.

Respondents who did not feel part of their local community had 80% higher odds of food insecurity than those who did (95% CI: 1.08–3.26). Participants classified as experiencing high social isolation on the composite score had two times the adjusted odds of food insecurity (AOR = 2.16, 95% CI: 1.09, 4.26) compared with those reporting low social isolation. On the sub-scale indicators of social support and connectedness, most associations remained significant after adjustment. Respondents who reported only occasionally having someone to share their feelings with had more than twice the odds of food insecurity (AOR = 2.41; 95% CI: 1.37–4.24). Those who frequently felt isolated from others (AOR = 2.91; 95% CI: 1.81–4.67), felt separate when with others (AOR = 2.73; 95% CI: 1.65–4.52), or felt alone and friendless (AOR = 3.13; 95% CI: 1.81–5.43) also had significantly increased odds of food insecurity. Additionally, difficulty getting in touch with others was associated with nearly double the odds of food insecurity (AOR = 1.99; 95% CI: 1.14–3.46). Only one measure, ‘finding it easy to relate to others’, did not reach statistical significance after adjustment (AOR = 1.64; 95% CI: 0.90–2.99), though the direction of association remained consistent.

## DISCUSSION

This study provides new evidence from regional Australia of strong and consistent associations between household food insecurity, poor mental health, and social isolation. Using survey data collected from the Illawarra and Shoalhaven regions, we found that nearly 40% of survey participants were food insecure and that those reporting poor mental health or lower levels of social connection had significantly higher odds of experiencing food insecurity. These findings contribute important insights to the limited literature on the social and psychological dimensions of food insecurity in regional Australian contexts and highlight an urgent need for integrated responses.

Our findings align with those of Seivwright *et al*. (2024), who also reported nearly 40% of their regional Australian study sample experienced food insecurity (Seivwright *et al*. 2024). However, given both studies over-representation of women and university-educated respondents, the absolute prevalence of food insecurity in these regional samples should be interpreted conservatively. Their study found that increasing food insecurity severity was associated with extreme financial coping strategies, such as reducing food intake and delaying medical care (Seivwright *et al*. 2024). Our study extends this work by highlighting the psychological and social problems associated with food insecurity, demonstrating that individuals with poor self-rated mental health or a diagnosed mental health condition had more than twice the odds of food insecurity. These findings support the growing consensus that food insecurity is not only a nutritional or economic concern, but also a key indicator of psychological vulnerability.

Consistent with previous international studies, we found that both self-rated poor mental health and diagnosed mental health conditions were independently associated with increased odds of food insecurity (Tarasuk *et al*. 2013, Aguiar *et al*. 2022, Jandaghian-Bidgoli *et al*. 2024). Participants who rated their mental health as fair-poor had more than twice the odds of being food insecure, while those with a diagnosed mental health issue (e.g. depression or anxiety) had similarly elevated odds. These findings align with another representative Australian study, which reported that adults with a mental illness were 1.4–1.9 times more likely to experience food insecurity across most mental health measures (Fry *et al*. 2025). A causal pathway between food insecurity and mental health outcomes has been investigated in recent longitudinal study (conducted in UK and France) that measured monthly food insecurity and mental health over a 1-year period. The authors reported finding within-person fluctuations in food insecurity that closely tracked changes in anxiety and depression, supporting a causal pathway (Bateson *et al*. 2025). While our cross-sectional design precludes causal inference, other longitudinal research suggests a bidirectional relationship (Bruening *et al*. 2017), where it is hypothesized that food insecurity may worsen mental health through chronic stress, stigma, and disrupted routines, while mental health challenges may impair a person’s ability to secure adequate food due to financial instability or difficulty accessing services (Park and Berkowitz 2025).

Our study also identified strong associations between food insecurity and multiple indicators of social isolation. Participants who felt isolated from others, friendless, or separate when around others were significantly more likely to be food insecure, even after controlling for key sociodemographic factors. Notably, there were strong interrelationships among social isolation variables, suggesting that social disconnection may operate as a unified construct rather than discrete experiences. These findings align with emerging research demonstrating the close relationship between food insecurity, loneliness, and weak social networks (Park and Berkowitz 2024). For example, Park and Berkowitz (2024) showed that experiencing food insecurity in over the previous year predicted increased social isolation and loneliness, alongside worsened mental health in US adults. Another study among adolescents from 39 countries reported a significantly higher prevalence of loneliness among those experiencing food insecurity (Wu *et al*. 2022). Again, a bi-directional mechanism of action may explain this link (McKenzie and Watts 2020). Social isolation may limit access to informal food-sharing networks or emotional support systems that can help buffer the effects of financial strain. Conversely, experiencing food insecurity is often accompanied by feelings of shame and stigma, and may lead individuals to withdraw from social interactions, reinforcing a cycle of disconnection and reduced support (Pineau *et al*. 2021). Positively, a UK FoodCycle study among community meal attendees found that while food poverty correlated with loneliness, participating in food-sharing activities fostered social connection among vulnerable community members (Rotenberg *et al*. 2021). Further, building social capital, including food-sharing and reciprocal resource exchange within communities, significantly improves food security (Nosratabadi *et al*. 2020).

The public health implications of these findings are significant. First, they reinforce the need to integrate both mental health and social wellbeing supports into food relief and nutrition programmes. Services addressing food insecurity should be trauma-informed and stigma-aware (Walker *et al*. 2023), and include referral pathways for psychological and social support (Escobar *et al*. 2021). Embedding mental health screening within food assistance programmes, coupled with social connection activities such as communal cooking or gardening, could address both material and psychosocial needs simultaneously.

Place-based initiatives in regional areas, such as community food hubs, could be designed to strengthen not only food access but also social connection and inclusion (Rose 2017). Community food hubs can use food provision as an entry point to link people with broader health and social services, including financial counselling, health checks, and mental health care. Evidence from the Illawarra and Shoalhaven shows that while community food hub users experience higher food insecurity and poorer health, they also report stronger community support, demonstrating how food can act as the ‘glue’ that connects people to wider systems of care (Kent *et al.*). Regional health services could build on these models by formalizing partnerships with food hubs and resourcing staff to deliver on-site supports such as nutrition education, primary health checks, and mental health referral pathways.

Second, our results point to the importance of tackling the underlying social and economic drivers of food insecurity, including low income, disability, and insecure housing, through policy reforms (Friel *et al*. 2015). A coordinated national approach could set measurable targets for reducing both food insecurity and loneliness, integrating them into the National Preventive Health Strategy and the Closing the Gap framework. Incorporating validated food insecurity and social isolation measures into national health surveys would allow progress to be tracked and interventions to be evaluated over time (Kent *et al*. 2025). A coordinated, whole-of-government strategy, including the establishment of a national Minister for Food, would improve accountability and drive intersectoral action across health, housing, employment, and community services (Carey *et al*. 2016). However, to enact federal policies within regional areas of Australia, partnership models are critical. Evidence from regional Australia shows that food security initiatives are most sustainable when organizations work together across sectors, combining resources, funding, and service delivery under shared goals (Godrich *et al*. 2025). Replicating and resourcing such cross-sectoral bodies nationally could provide the infrastructure required for coordinated place-based responses (Nelson *et al*. 2025).

The strengths of this study include the use of a validated, multi-item measure of food insecurity, the 18-item Household Food Security Survey Module, which captures a continuum of severity and has been widely used in Australian and international research (Bickel *et al*. 2000, Baker *et al*. 2024). Additionally, validated self-report measures of physical and mental health were employed, consistent with prior studies linking subjective health status to broader wellbeing outcomes (Lundberg and Manderbacka 1996). Their inclusion provides a pragmatic method for capturing subjective health perceptions in large-scale surveys, especially when objective clinical measures are not feasible. However, some limitations should also be noted. Given the cross-sectional design, temporality cannot be established, and causality cannot be inferred. The observed relationships are plausibly bidirectional, whereby food insecurity may worsen mental health and social connection, while poor mental health and social isolation may increase vulnerability to food insecurity. While the flyer distribution aimed to reach residents beyond existing digital and social networks, the overall approach may still have led to over-representation of individuals who are more socially connected or engaged with community channels. This potential recruitment bias should be considered when interpreting the generalisability of findings. Furthermore, the survey sample was disproportionately female and highly educated, which may have led to an underestimation of food insecurity prevalence and limit generalisability by providing an incomplete representation of underrepresented groups such as those with lower education levels, or individuals from culturally and linguistically diverse backgrounds. While the use of online recruitment facilitated broad participation, it may have excluded individuals with limited digital access or literacy, who are often at greater risk of food insecurity. Future studies should use mixed recruitment methods and partner with community organizations and services that already engage with vulnerable populations to improve reach and inclusivity. In addition, our study was unable to examine how co-occurring poor mental and physical health influenced the risk and severity of food insecurity due to limited subgroup sample sizes. Future research should prioritize this area to identify particularly vulnerable populations and inform integrated interventions. Larger, longitudinal studies are needed to clarify causal pathways and assess whether poor health and food insecurity act synergistically.

## CONCLUSION

This study highlights the interconnectedness of food insecurity, mental health, and social isolation in a regional Australian setting. Findings point to the need for holistic, multisectoral responses that address both the material and social dimensions of food insecurity. Future research should explore longitudinal and intervention-based designs to better understand causal pathways and to evaluate the impact of integrated public health strategies.

## Supplementary Material

daaf176_Supplementary_Data

## Data Availability

The data underlying this article will be shared on reasonable request to the corresponding author.

## References

[daaf176-B1] Aguiar A, Pinto M, Duarte R. The bad, the ugly and the monster behind the mirror—food insecurity, mental health and socio-economic determinants. J Psychosom Res 2022;154:110727. 10.1016/j.jpsychores.2022.110727 35086053

[daaf176-B2] Baker S, Gallegos D, Rebuli MA et al Food insecurity screening in high-income countries, tool validity, and implementation: a scoping review. Nutrients 2024;16:1684. 10.3390/nu16111684 38892619 PMC11174716

[daaf176-B3] Barton J, Osuagwu UL, Cockrell-Reed K et al Factors associated with loneliness in rural Australia: a web-based cross-sectional survey. Soc Sci Humanit Open 2024;10:101154. 10.1016/j.ssaho.2024.101154

[daaf176-B4] Bateson M, Chevallier C, Johnson EA et al Does food insecurity cause anxiety and depression? Evidence from the changing cost of living study. PLoS Ment Health 2025;2:e0000320. 10.1371/journal.pmen.0000320 PMC12798610 41661973

[daaf176-B5] Bickel G, Nord M, Price C et al Guide to measuring household food security, revised 2000. 2000. https://nhis.ipums.org/nhis/resources/FSGuide.pdf

[daaf176-B6] Bombak AE . Self-rated health and public health: a critical perspective. Front Public Health 2013;1:15. 10.3389/fpubh.2013.00015 24350184 PMC3855002

[daaf176-B7] Bruening M, Dinour LM, Chavez JBR. Food insecurity and emotional health in the USA: a systematic narrative review of longitudinal research. Public Health Nutr 2017;20:3200–8. 10.1017/S1368980017002221 28903785 PMC10261670

[daaf176-B8] Carey R, Caraher M, Lawrence M et al Opportunities and challenges in developing a whole-of-government national food and nutrition policy: lessons from Australia’s national food plan. Public Health Nutr 2016;19:3–14. 10.1017/S1368980015001834 26073889 PMC10271130

[daaf176-B9] Compton MT . Food insecurity as a social determinant of mental health. Psychiatr Ann 2014;44:46–51. 10.3928/00485713-20140108-08

[daaf176-B10] Escobar ER, Pathak S, Blanchard CM. Screening and referral care delivery services and unmet health-related social needs: a systematic review. Prev Chronic Dis 2021;18:E78. 10.5888/pcd18.200569 34387188 PMC8388203

[daaf176-B11] Foodbank Australia . Foodbank hunger report 2023. 2023. https://reports.foodbank.org.au/foodbank-hunger-report-2024/

[daaf176-B12] Friel S, Hattersley L, Ford L et al Evidence review: addressing the social determinants of inequities in healthy eating. In: The National Centre for Epidemiology and Population Health. Canberra: The Australian National University, 2015.

[daaf176-B13] Fry JM, Temple JB, Williams R. Food insecurity and health conditions in the Australian adult population: a nationally representative analysis. Nutr Diet 2025;82:64–75. 10.1111/1747-0080.12907 39429055 PMC11795227

[daaf176-B14] Gallegos D, Booth S, Pollard CM et al Food security definition, measures and advocacy priorities in high-income countries: a Delphi consensus study. Public Health Nutr 2023;26:1986–96. 10.1017/S1368980023000915 37144401 PMC10564592

[daaf176-B15] Godrich SL, Stoneham M, Chiera I et al Increasing the effectiveness of rural, regional and remote food security initiatives through place-based partnerships-a qualitative study. Health Promot J Austr 2025;36:e70048. 10.1002/hpja.70048 40304187 PMC12042255

[daaf176-B16] Gonyea JG, O'Donnell AE, Curley A et al Food insecurity and loneliness amongst older urban subsidised housing residents: the importance of social connectedness. Health Soc Care Community 2022;30:e5959–67. 10.1111/hsc.14027 36124722

[daaf176-B17] Graham C, Ciciurkaite G. The risk for food insecurity and suicide ideation among young adults in the United States: the mediating roles of perceived stress and social isolation. Soc Ment Health 2023;13:61–78. 10.1177/21568693221120066

[daaf176-B18] Gundersen C, Ziliak JP. Food insecurity and health outcomes. Health Aff (Millwood) 2015;34:1830–9. 10.1377/hlthaff.2015.0645 26526240

[daaf176-B19] Hill CM, Tseng AS, Holzhauer K et al Association between health care access and food insecurity among lower-income older adults with multiple chronic conditions in Washington state, USA. Public Health Nutr 2023;26:199–207. 10.1017/S1368980022001240 35603699 PMC11077446

[daaf176-B20] Jandaghian-Bidgoli M, Kazemian E, Shaterian N et al Focusing attention on the important association between food insecurity and psychological distress: a systematic review and meta-analysis. BMC Nutr 2024;10:118. 10.1186/s40795-024-00922-1 39243085 PMC11378639

[daaf176-B21] Jones AD . Food insecurity and mental health status: a global analysis of 149 countries. Am J Prev Med 2017;53:264–73. 10.1016/j.amepre.2017.04.008 28457747

[daaf176-B22] Kent K, Charlton K, Andrews K et al Food is the Glue: Community Centres in the Illawarra and Shoalhaven Use Food as a Gateway to Supporting Wellbeing & Connection. University of Wollongong. Report. 10.71747/uowr3gk326m.28509653.v1

[daaf176-B23] Kent K, Murray S, Penrose B et al The new normal for food insecurity? A repeated cross-sectional survey over 1 year during the COVID-19 pandemic in Australia. Int J Behav Nutr Phys Act 2022;19:115. 10.1186/s12966-022-01347-4 36068538 PMC9449271

[daaf176-B24] Kent K, Kleve S, Lindberg R. Two minutes is all it takes: measuring food security in Australia. 2025. https://thepolicymaker.jmi.org.au/two-minutes-is-all-it-takes-measuring-food-security-in-australia/ (10 September 2025, date last accessed).

[daaf176-B25] Lee K . Household marginal food security is associated with poorer self-rated health in Korean adults. Nutr Res 2022;100:33–41. 10.1016/j.nutres.2022.01.001 35124552

[daaf176-B26] Lundberg O, Manderbacka K. Assessing reliability of a measure of self-rated health. Scand J Soc Med 1996;24:218–24. 10.1177/140349489602400314 8878376

[daaf176-B27] McKenzie JS, Watts DC. Re-thinking the relationship between food insecurity, health and social isolation. Proc Nutr Soc 2020;79:E740. 10.1017/S0029665120007260

[daaf176-B28] Martin MS, Maddocks E, Chen Y et al Food insecurity and mental illness: disproportionate impacts in the context of perceived stress and social isolation. Public Health 2016;132:86–91. 10.1016/j.puhe.2015.11.014 26795678

[daaf176-B29] Men F, Gundersen C, Urquia ML et al Food insecurity is associated with higher health care use and costs among Canadian adults. Health Aff (Millwood) 2020;39:1377–85. 10.1377/hlthaff.2019.01637 32744947

[daaf176-B30] Mungai NW, Priestly J, Pawar M. Food insecurity in regional rural Australia. Aust Soc Work 2020;73:149–61. 10.1080/0312407X.2019.1662820

[daaf176-B300] Nelson R, Lim-Camacho L, Robinson CJ (eds.) *Towards a State of the Food System Report for Australia*. Australia: CSIRO, 2025.

[daaf176-B31] Nosratabadi S, Khazami N, Abdallah MB et al Social capital contributions to food security: a comprehensive literature review. Foods 2020;9:1650. 10.3390/foods9111650 33198127 PMC7698312

[daaf176-B32] Park S, Berkowitz SA. Social isolation, loneliness, and quality of life among food-insecure adults. Am J Prev Med 2024;67:120–3. 10.1016/j.amepre.2024.02.001 38331116

[daaf176-B33] Park S, Berkowitz SA. Association of food insecurity with mental health status, mental health services utilisation and general healthcare utilisation among US adults. J Epidemiol Community Health 2025;79:332–9. 10.1136/jech-2024-221900 39603685

[daaf176-B34] Pineau C, Williams PL, Brady J et al Exploring experiences of food insecurity, stigma, social exclusion, and shame among women in high-income countries: a narrative review. Food Stud 2021;8. 10.15353/cfs-rcea.v8i3.473

[daaf176-B35] Plante C, Missiuna S, Neudorf C. The validity and reliability of dichotomized self-rated health under different cutpoints. medRxiv, 10.1101/2024.04.18.24306035, 19 April 2024, preprint: not peer reviewed.

[daaf176-B36] Pollard CM, Booth S. Food insecurity and hunger in rich countries—it is time for action against inequality. Int J Environ Res Public Health 2019;16:1804. 10.3390/ijerph16101804 31117216 PMC6572174

[daaf176-B37] Pourmotabbed A, Moradi S, Babaei A et al Food insecurity and mental health: a systematic review and meta-analysis. Public Health Nutr 2020;23:1778–90. 10.1017/S136898001900435X 32174292 PMC10200655

[daaf176-B38] Rose N . Community food hubs: an economic and social justice model for regional Australia? Rural Soc 2017;26:225–37. 10.1080/10371656.2017.1364482

[daaf176-B39] Rotenberg K, Surman E, McGrath M. Loneliness, food poverty, and perceived benefits of communal food consumption from a charity service. J Poverty 2021;25:465–79. 10.1080/10875549.2020.1869667

[daaf176-B40] Seivwright A, Kocar S, Visentin D et al Cutting more than meals: increasing severity of food insecurity is associated with the number and types of household financial strategies used to cope with inflation. Aust J Soc Issues 2024;59:65–86. 10.1002/ajs4.314

[daaf176-B41] Tarasuk V, Mitchell A, McLaren L et al Chronic physical and mental health conditions among adults may increase vulnerability to household food insecurity. J Nutr 2013;143:1785–93. 10.3945/jn.113.178483 23986364

[daaf176-B42] Terwee CB, Bot SDM, de Boer MR et al Quality criteria were proposed for measurement properties of health status questionnaires. J Clin Epidemiol 2007;60:34–42. 10.1016/j.jclinepi.2006.03.012 17161752

[daaf176-B43] Walker C, Klein O, Schan H et al Hunger trauma, relational care and emergency food support. In: Boden-Stuart Z, Larkin M (eds.), Relationships and Mental Health: Relational Experience in Distress and Recovery. Springer International Publishing, 2023, 179–95.

[daaf176-B44] Weaver LJ, Fasel CB. A systematic review of the literature on the relationships between chronic diseases and food insecurity. Food Nutr Sci 2018;9:519. 10.4236/fns.2018.95040

[daaf176-B45] Wu H, Gu Z, Zeng L et al Do global adolescents with food insecurity feel lonely? Front Public Health 2022;10:820444. 10.3389/fpubh.2022.820444 35223740 PMC8868937

