# Supplementary material for: Hungry for connection: associations between social isolation, mental health, and food insecurity in regional Australian adults
Source: Health Promot Int. 2025 Oct 24;40(5):daaf176. doi: 10.1093/heapro/daaf176 (PMC12550559; doi:10.1093/heapro/daaf176)
Supplement: daaf176_Supplementary_Data [file daaf176_supplementary_data.docx]

**Supplementary Table 1.**Correlation matrix between Health, Socio-Demographic and Food insecurity variables.

|  |  | Food Insecurity | A | B | C | D | E | F | G | H | I | J | K | L | M | N | O | P | Q | R | S |
| --- | --- | --- | --- | --- | --- | --- | --- | --- | --- | --- | --- | --- | --- | --- | --- | --- | --- | --- | --- | --- | --- |
| A | Self-rated Mental Health | 0.349^**^ | -- |  |  |  |  |  |  |  |  |  |  |  |  |  |  |  |  |  |  |
| B | Self-rated Physical Health | 0.316^**^ | 0.567^**^ | -- |  |  |  |  |  |  |  |  |  |  |  |  |  |  |  |  |  |
| C | Diagnosed Mental Health Issue | -0.320^**^ | -0.533^**^ | -0.284^**^ | -- |  |  |  |  |  |  |  |  |  |  |  |  |  |  |  |  |
| D | Felt part of local community | 0.220^**^ | 0.301^**^ | 0.232^**^ | -0.219^**^ | -- |  |  |  |  |  |  |  |  |  |  |  |  |  |  |  |
| E | Easy to relate to others | 0.276^**^ | 0.473^**^ | 0.272^**^ | -0.290^**^ | 0.437^**^ | -- |  |  |  |  |  |  |  |  |  |  |  |  |  |  |
| F | Has someone to share feelings with | 0.252^**^ | 0.353^**^ | 0.263^**^ | -0.194^**^ | 0.374^**^ | 0.632^**^ | -- |  |  |  |  |  |  |  |  |  |  |  |  |  |
| G | Felt isolated from others | -0.344^**^ | -0.490^**^ | -0.336^**^ | 0.324^**^ | -0.388^**^ | -0.542^**^ | -0.510^**^ | -- |  |  |  |  |  |  |  |  |  |  |  |  |
| H | Found it easy to get in touch with others | 0.269^**^ | 0.352^**^ | 0.231^**^ | -0.193^**^ | 0.364^**^ | 0.603^**^ | 0.659^**^ | -0.486^**^ | -- |  |  |  |  |  |  |  |  |  |  |  |
| I | Felt separate from others | -0.317^**^ | -0.466^**^ | -0.331^**^ | 0.315^**^ | -0.339^**^ | -0.537^**^ | -0.442^**^ | 0.660^**^ | -0.386^**^ | -- |  |  |  |  |  |  |  |  |  |  |
| J | Felt alone and friendless | -0.318^**^ | -0.445^**^ | -0.339^**^ | 0.285^**^ | -0.357^**^ | -0.480^**^ | -0.514^**^ | 0.698^**^ | -0.483^**^ | 0.667^**^ | -- |  |  |  |  |  |  |  |  |  |
| K | Gender | -0.069 | 0.002 | -0.046 | -0.060 | -0.004 | -0.157^**^ | -0.170^**^ | 0.044 | -0.115^**^ | 0.083^*^ | 0.100^*^ | -- |  |  |  |  |  |  |  |  |
| L | Age | -0.221^**^ | -0.242^**^ | 0.045 | 0.171^**^ | -0.155^**^ | -0.201^**^ | 0.064 | 0.157^**^ | -0.091^*^ | 0.123^**^ | 0.103^*^ | -0.029 | -- |  |  |  |  |  |  |  |
| M | Education | -0.239^**^ | -0.076 | -0.185^**^ | 0.116^**^ | -0.069 | -0.139^**^ | -0.211^**^ | 0.134^**^ | -0.198^**^ | 0.101^*^ | 0.133^**^ | 0.080 | -0.160^**^ | -- |  |  |  |  |  |  |
| N | Aboriginal and/or Torres Strait Islander status | 0.247^**^ | 0.078 | 0.090^*^ | -0.089^*^ | 0.015 | 0.095^*^ | 0.163^**^ | -0.106^**^ | 0.080^*^ | -0.069 | -0.075 | -0.018 | -0.056 | -0.211^**^ | -- |  |  |  |  |  |
| O | Disability | 0.199^**^ | 0.274^**^ | 0.454^**^ | -0.207^**^ | 0.115^**^ | 0.146^**^ | 0.213^**^ | -0.200^**^ | 0.159^**^ | -0.169^**^ | -0.199^**^ | 0.014 | 0.214^**^ | -0.121^**^ | 0.075 | -- |  |  |  |  |
| P | Born in Australia | 0.058 | 0.083^*^ | 0.068 | -0.091^*^ | 0.013 | -0.070 | -0.077 | -0.017 | -0.071 | 0.015 | 0.031 | 0.050 | -0.180^**^ | 0.006 | 0.092^*^ | 0.067 | -- |  |  |  |
| Q | Language other than English | 0.157^**^ | 0.015 | 0.001 | 0.021 | -0.050 | 0.092^*^ | 0.040 | -0.045 | 0.094^*^ | -0.068 | -0.047 | -0.043 | -0.042 | 0.051 | 0.135^**^ | 0.025 | -0.198^**^ | -- |  |  |
| R | Household Composition | 0.127^**^ | 0.031 | 0.003 | -0.034 | -0.041 | 0.018 | -0.072 | 0.042 | -0.005 | 0.020 | 0.017 | 0.089^*^ | -0.187^**^ | 0.108^**^ | 0.167^**^ | -0.007 | 0.005 | 0.124^**^ | -- |  |
| S | Employment | 0.132^**^ | 0.045 | 0.146^**^ | -0.031 | -0.017 | 0.080^*^ | 0.123^**^ | -0.139^**^ | 0.137^**^ | -0.131^**^ | -0.126^**^ | 0.051 | 0.210^**^ | -0.254^**^ | 0.035 | 0.196^**^ | -0.064 | 0.048 | -0.103^**^ | -- |
| B | Income | -0.235^**^ | -0.113^**^ | -0.152^**^ | 0.102^*^ | -0.057 | -0.058 | -0.150^**^ | 0.101^*^ | -0.080^*^ | 0.151^**^ | 0.145^**^ | 0.015 | -0.025 | 0.212^**^ | -0.011 | -0.176^**^ | 0.008 | -0.087^*^ | 0.130^**^ | -0.287^**^ |

**. Correlation is significant at the 0.01 level (2-tailed). *. Correlation is significant at the 0.05 level (2-tailed
